# Anti-glaucoma agents-induced pseudodendritic keratitis presumed to be herpetic simplex keratitis: a clinical case series

**DOI:** 10.1038/s41598-021-01073-0

**Published:** 2021-11-02

**Authors:** Huai-Lung Chang, Bo-I Kuo, Jo-Hsuan Wu, Wei-Lun Huang, Chien-Chia Su, Wei-Li Chen

**Affiliations:** 1 Department of Ophthalmology, National Taiwan University Hospital, No. 7, Chung-Shan South Road, Taipei, Taiwan; 2 Section of Ophthalmology, National Taiwan University Cancer Center, Taipei, Taiwan; 3 Shiley Eye Institute and Viterbi Family Department of Ophthalmology, Hamilton Glaucoma Center, University of California, San Diego, CA USA; 4 Graduate Institute of Clinical Medicine, National Taiwan University College of Medicine, Taipei, Taiwan; 5 Advanced Ocular Surface and Corneal Nerve Regeneration Center, National Taiwan University Hospital, Taipei, Taiwan; 6 Department of Ophthalmology, National Taiwan University College of Medicine, Taipei, Taiwan

**Keywords:** Medical research, Outcomes research

## Abstract

Anti-glaucoma agents-induced corneal toxicity may be misdiagnosed as herpetic simplex keratitis (HSK). In our study, nineteen glaucoma patients were presumed to have HSK before referral. Corneal lesions were classified into (I) linear pseudodendritic lesions formed by elevated opacified cells, (II) linear pseudodendritic lesions formed by grouped superficial punctate keratitis (SPK), (III) satellite full-thickness epithelial defects, (IV) satellite lesions formed by elevated opacified cells, and (V) geographic lesions formed by grouped SPK. We observed thirty-one events, with 15 in the lower and 16 in the central corneas. There were 21 (67.7%) type II, five (16.1%) type V, two (6.5%) of each for types III and IV, and one (3.2%) type I events. Among linear lesions (types I and II), 17 (77.3%) had horizontal and 5 (22.7%) had curvilinear orientations. Exposure duration to the last-added anti-glaucoma agent was three days to 14.5 years. About half of the events (16/31, 51.6%) used prostaglandin analogues, and 30/31 (96.8%) applied benzalkonium chloride (BAK)-containing agents. All lesions resolved within two months after decreasing offending medications or enhancing protection of ocular surface. In conclusion, anti-glaucoma agents-induced pseudodendritic keratitis presents majorly in central-lower cornea as horizontally linear lesions, and BAK-containing agents are observed in the most events.

## Introduction

Glaucoma is a major cause of irreversible blindness, estimated to involve almost 111 million people in 2040 worldwide^1,2^, and anti-glaucoma agents are currently the most common treatment strategies. Despite the effects of lowering intraocular pressure (IOP)^3–5^, ocular toxicity of these agents is frequently reported, including subconjunctival fibrosis, allergy, decreased tear film, and superficial corneal toxicity^6–10^. Daily and repetitive exposure of ocular surface to the active compounds or the preservatives in the topical anti-glaucoma medications may be deleterious to ocular surface. The most commonly applied preservative in glaucoma medications is benzalkonium chloride (BAK), and had been reported to interrupt the corneal epithelial barrier function with its detergent properties and damage the corneal nerves, as well as disrupting the production and stabilization of tear film^11–14^. Although the evolving fixed combination drugs^15^ and new surgical techniques^16,17^ can mitigate drug application frequency and associated ocular surface damage, many glaucoma patients are still receiving multi-eye drop treatments. The intricate medication history may confound ophthalmologists and complicate the diagnosis of ocular surface diseases in these patients.

Among the possible ocular surface complications in patients receiving anti-glaucoma agents, herpetic simplex keratitis (HSK) is one of the most severe and had been emphasized in patients receiving topical prostaglandins. After the first announcement of this complication of prostaglandins in 1999^18^, several reports have been published^19–24^. Awareness of this side effect is important since HSK can be managed with antiviral medications, while significant visual impairments may occur if left untreated^25,26^. However, over-alert of this complication may lead to misdiagnosis in patients presenting with similar corneal lesions of other causes, especially those with pseudodendritic lesions caused by certain anti-glaucoma agents^23,27^. Since treatment strategies for HSK and medication-induced corneal toxicity are different, distinguishing them is essential.

To the best of our knowledge, there has not been a large-scaled case series analysing the corneal lesions caused by anti-glaucoma agents that were initially misdiagnosed as HSK. We enrolled 19 patients diagnosed with anti-glaucoma agents-induced pseudodendritic keratitis in tertiary medical center with the presumed diagnosis of HSK in primary care clinics before referral. Based on the lesion morphology and medication history, a simple reference guideline to ameliorate identification of this disease was provided.

## Methods

In this 6-year retrospective, non-comparative, non-interventional case series study, we enrolled patients referred to our hospital from January, 2015 to January, 2021 with the presumed diagnosis of HSK made by different primary care doctors. The study was approved by the Institutional Review Board of NTUH (202007139RIND) as per the tenets of the Declaration of Helsinki. Informed consent was obtained from all subjects for utilizing their information and de-identified images for study and publication.

### Criteria for subject inclusion

All patients were immunocompetent and had been under topical anti-glaucoma agents unilaterally or bilaterally before referral. They were all diagnosed with HSK by different referral doctors, with symptoms including ocular pain, foreign body sensations, blurred visions, etc. None had ocular surgery within one year prior to the presumed diagnosis of HSK. Detailed history of topical anti-glaucoma agents (drug species and duration), demographic information, past ocular history, external eye photos, and treatment course before and after referral were obtained.

The topical medications used in this study included dorzolamide 2% (Trusopt, Merk & Co., Onv., Whitehouse Station, NJ, US, with 0.0075% BAK), latanoprost 0.005% (Xalatan, Pfizer, New York, NY, US, with 0.02% BAK), brimonidine 0.15% (Alphagan P, Allergan, Irvine, CA, US with 0.005% purite), carteolol 2% (Mikelan, Otsuka, Tokyo, Japan, with 0.005% BAK), timolol 0.5% (Timoptic, Merk & Co. White house station, NJ, US, with 0.01% BAK), pilocarpine 2% (Isopto Carpine, Alcon, Fort Worth, TX, USA, with 0.01% BAK), preservative free bimatoprost 0.03% (Lumigan, Allergan, Madison, NJ, US), and dorzolamide 2%/timolol 0.5% combination (Cosopt, Merck & Co., Inc., Whitehouse Station, NJ, US, with 0.0075% BAK).

### Confirmation of non-HSK pseudodendritic keratitis

To confirm the HSK-unrelated nature of the lesions, all patients were examined by the following criteria: (1) improvement of corneal lesions after removing the presumed triggering medications or applying ocular surface protecting methods, including topical lubricants or therapeutic soft contact lens (TSCL) without using antiviral medications, (2) no recurrence of the corneal lesions after removing the presumed triggering medications, (3) absences of characteristics corneal lesions of HSV, such as dendritic lesions with terminal bulbs, (4) negative viral culture results of the lesions, and (5) bilateral involvement, which is less likely HSK if the patient is immunocompetent. The prevalence of bilateral HSK is estimated to be 1.3–12% in the immunocompetent adults^28,29^. To confirm the diagnosis of a non-HSK pseudodendritic keratitis, it is required that subjects should meet rules one to three, and may be further confirmed by the addition of either rules four or five.

### Analysis of the corneal lesions

Since some patients had recurrent diseases, the corneal lesions were counted based on “event,” defined as the occurrence of lesions per time in per eye. Bilateral involvement at the same time was identified as two events, and lesions presenting in the same eye during separated periods were counted as individual events. Meanwhile, the “episode” was defined as the occurrence of lesions per time in per patient, with bilateral involvement at the same time identified as one episode.

**Figure 1:**
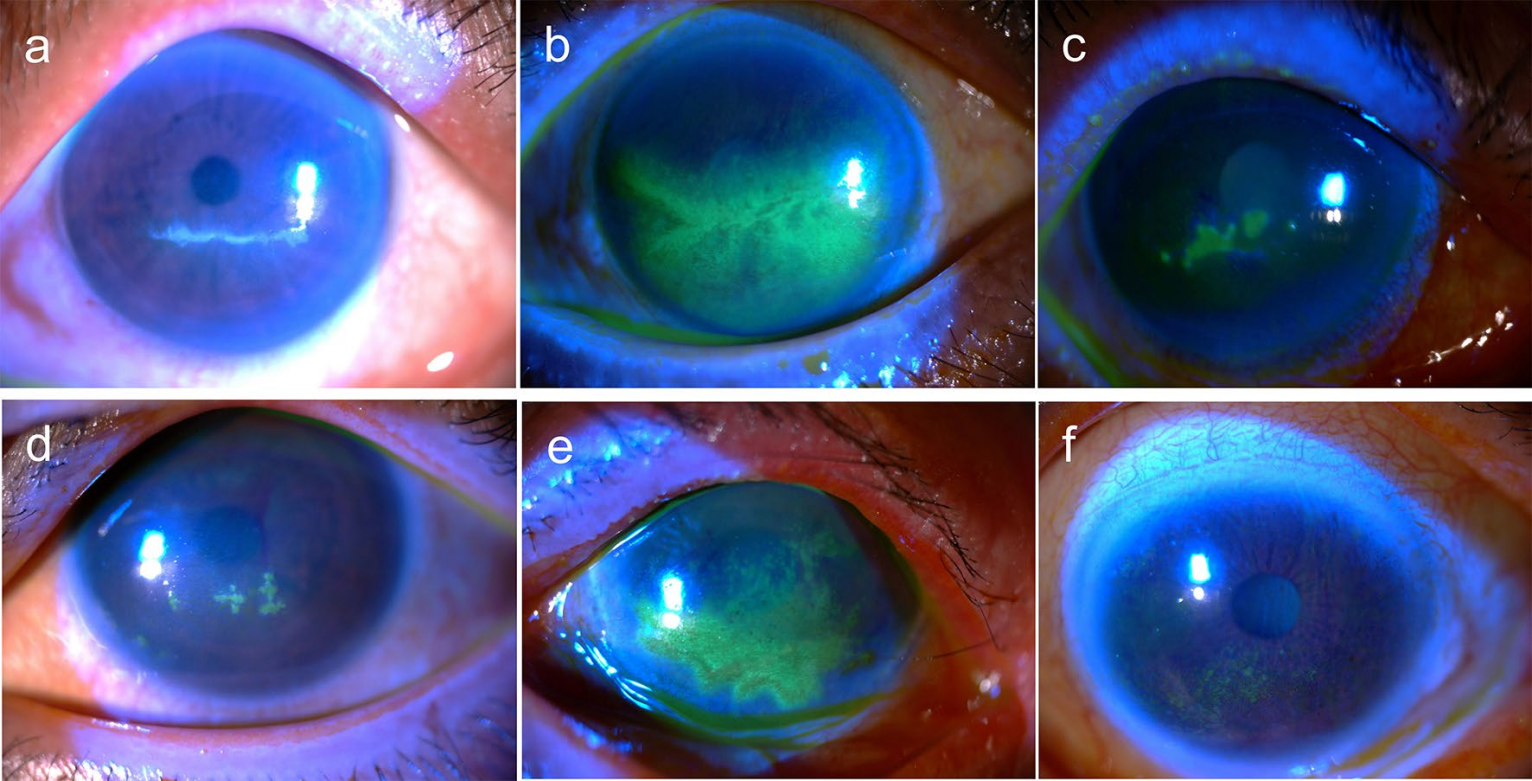
Examples of type I–V anti-glaucoma agent-induced pseudodendritic lesions, in comparison with typical superficial punctate keratitis (SPK). (**a**) Type I. Linear pseudodendritic lesions with elevated opacified cells. (**b**) Type II. Linear pseudodendritic lesions formed by grouped superficial punctate keratitis. (**c**) Type III. Satellite full thickness epithelial defects. (**d**) Type IV. Satellite lesions formed by elevated opacified cells. (**e**) Type V. Geographic lesions formed by grouped superficial punctate keratitis. (**f**) Typical superficial punctate keratitis in patients with dye eye diseases (DED) without pseudodendritic presentation.

**Figure 2:**
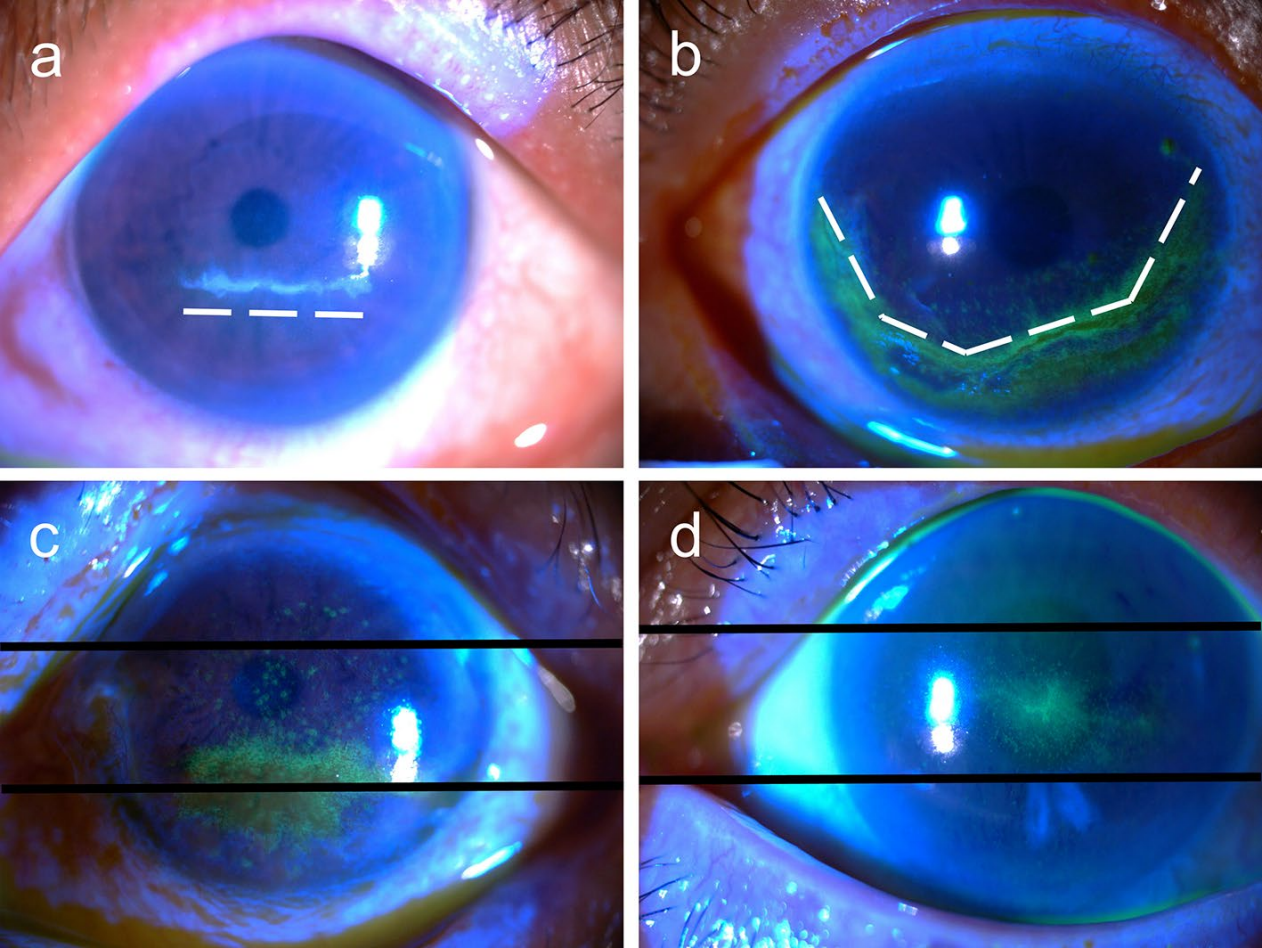
Examples of the orientation and location of anti-glaucoma agent-induced pseudodendritic lesions. The orientation was only measured in linear lesions (type I and II). The white dashed lines illustrate the contour of corneal lesions. The black solid lines divided the corneas as three equal parts. (**a**) Horizontal orientation. (**b**) Curvilinear orientation. (**c**) Lower-located pseudodendritic lesions, with more than 2/3 of the lesion involved the lower cornea. (**d**) Central-located pseudodendritic lesions, with more than 2/3 of the lesions involving the central cornea.

The lesions were classified into five types based on morphological presentations (Fig. 1), including (I) linear pseudodendritic lesions formed by elevated opacified cells, (II) linear pseudodendritic lesions formed by grouped superficial punctate keratitis (SPK), (III) satellite full-thickness epithelial defects, (IV) satellite lesions formed by elevated opacified cells, and (V) geographic ulcers formed by grouped SPK. Characteristics of HSK, including terminal bulbs and central fluorescein stains, were not present. The cornea was divided into upper, central, and lower thirds. Locations of the lesions were based on the majorly involved areas (> 2/3 of the lesion area). The linear lesions (types I and II) were divided into horizontal and curvilinear based on their orientations. Examples of different locations and orientations were shown in Fig. 2.

## Results

### Characteristics of subjects

A total of 22 episodes, 31 events of 19 patients were included. Characteristics of the participants were in Table 2. Mean age of the patients was 72.5 ± 15.6 years. Sixteen (84.2%) patients were older than 65 years old. Male patients were predominant (14, 73.7%).

Among the 19 participants, three (15.8%) had type 2 diabetes mellitus, two (10.5%) had Sjogren’s syndrome (SS) with dry eye disease (DED), and nine (47.4%) had non-Sjogren’s DED characterized by decreased tear-film breakup time, decreased tear film meniscus or positive Schirmer’s test (Table 2).

### Characteristics of the pseudodendritic lesions

Most patients experienced single episode, while three patients had two episodes. Thirteen (59.1%) of the 22 episodes were unilateral and the other nine (40.9%) were bilateral, contributing to 13 and 18 events, respectively. Among the unilateral episodes, two had unilateral eye drop application and 11 had bilateral eye drop treatment. Among those using bilateral eye drops, case 2 administered additional topical pilocarpine to the diseased eye, while all others applied same medications in both eyes. In the nine bilateral episodes, all patients administered same anti-glaucoma agents in both eyes.

To exclude HSK, HSV viral culture was performed in the first seven patients (cases 1 to 7) and elicited negative results. The rest patients were not tested for HSV and was examined using the aforementioned criteria, which we believed was sufficient for differentiation.

All 31 events were classified by lesion locations, types of morphological presentation, and lesion orientations. About half (16, 51.6%) of the events were found located in the lower cornea and the other half (15, 48.4%) were in the central cornea. None was in the upper cornea. For the types of morphological presentation, there are 21 (67.7%) type II, five (16.1%) type V, two (6.5%) of each for types III and IV, and one (3.2%) type I events. Among the 22 linear events (types I and II), 17 (77.3%) were horizontal in orientation and five (22.7%) were curvilinear. No vertical linear lesion was noted. For the three patients that experienced two episodes, there were changes in lesion morphology during the recurrent episodes. In the first episodes, case 8 had horizontal, central type I lesion in the left eye, case 10 had horizontal, central type II lesion in both eyes, and case 16 had central type III lesion in the right eye. In the recurrent episodes, case 8 had central type III lesion in the right eye, case 10 had horizontal, central type II lesion in the left eye, and case 16 had lower type II lesions with curvilinear orientation in the right eye and horizontal orientation in the left eye.

### Medication history of applying anti-glaucoma agents

In our study, 19 (61.2%) of the 31 events used single anti-glaucoma medication and 12 (38.7%) used two medications. No patient received over two drugs. The most frequently used long-term medication in our study was latanoprost (10, 38.5%), followed by carteolol (5, 19.2%), brimonidine (4, 15.4%) and dorzolamide/timolol (4, 15.4%). The last-added meditations included dorzolamide (2, 33.3%), latanoprost (2, 33.3%), carteolol (1, 16.7%) and dorzolamied/timolol (1, 16.7%). Although it is difficult to conclude whether the active compounds or preservatives in the drugs contributed to corneal toxicity, BAK was found in most of the medications in this study. A total of eight kinds of anti-glaucoma agents were used in our patients, in which seven were applied before corneal lesions appeared. BAK preservative was found in six (85.7%) of the seven medications, except for brimonidine, which contained purite as preservative. Preservative-free bimatoprost was used as the alternative anti-glaucoma agent after corneal lesions appeared (cases 3, 9–13, 19). BAK-containing medications were used in 30 (96.8%) out of the 31 events (Tables 1 and 2).

**Table 1:** Demographic and clinical characteristics of study participants.

Characteristics	No. (%)^a^, mean ± SD, or range
**Age (years)**	
Mean	72.5 ± 15.6
Range	26–96
< 65	3 (15.8)
≥ 65	16 (84.2)
**Gender**	
Male	14 (73.7)
Female	5 (26.3)
**Past medical history**	
DM	3 (15.8)
DED	9 (47.4)
SS	2 (10.5)
Atopic dermatitis	1 (5.3)
**Anti-glaucoma agents (preservative)**	
Long-term medications	
Latanoprost (BAK)	10 (38.5)
Carteolol (BAK)	5 (19.2)
Brimonidine (purite)	4 (15.4)
Dorzolamide/Timolol (BAK)	4 (15.4)
Timolol (BAK)	2 (7.7)
Pilocarpine (BAK)	1 (3.8)
Last-added mediations	
Dorzolamide (BAK)	2 (33.3)
Latanoprost (BAK)	2 (33.3)
Carteolol (BAK)	1 (16.7)
Dorzolamide/Timolol (BAK)	1 (16.7)
**Durations of anti-glaucoma agents usage**	
Long-term medications	
≤ 3 months	4 (18.2)
> 3 months, ≤ 1 year	8 (36.4)
> 1 year, ≤ 5 years	6 (27.3)
> 5 years	4 (18.2)
Last-added medications	
≤ 1 month	3 (50)
> 1 month	3 (50)
Types of pseudodendritic lesions	
I	1 (3.2)
II	21 (67.7)
III	2 (6.5)
IV	2 (6.5)
V	5 (16.1)
Locations of pseudodendritic lesions	
Upper	0
Center	16 (51.6)
Lower	15 (48.4)
Orientations of linear pseudodendritic lesions (type I and II lesions)	
Horizontal	17 (77.3)
Curvilinear	5 (22.7)

*DM* diabetes mellitus, *DED* non-Sjogren dry eye disease, *SS* Sjogren syndrome.

^a^The No. for age, gender and past medical history refers to the numbers of patients for each characteristic. The No. for anti-glaucoma agents refers to the numbers of episodes that each medication was used in. The No. for types and locations of pseudodendritic lesions, and orientations of linear pseudodendritic lesions refers to the numbers of events for each characteristic.

**Table 2:** Brief history of the case series.

Case	Age	Gender	Past history	Episode	Corneal lesion, laterality	Long-term medication (preservatives)/laterality	Duration	Last-added medication (preservatives)/laterality	Duration	HSV culture	Management^a^
1	26	M	DED	1st	Type IV, ou	Timolol (BAK)/ou	1 year	NA		Negative	A, B
2	67	M	DED	1st	Type II, od	Pilocarpine (BAK)/od	1 year	Dorzolamide (BAK)/ou	1 month	Negative	C
3	60	F	DM	1st	Type II, os	Latanoprost (BAK)/ou	14 years	NA		Negative	A, F
4	70	F	–	1st	Type II, os	Latanoprost (BAK)/ou	6 years	NA		Negative	A, C, E
5	93	M	DED	1st	Type V, ou	Dorzolamide/timolol (BAK)/ou	1 year	NA		Negative	A
6	75	M	DED	1st	Type II, ou	Carteolol (BAK)/ou	7 years	Latanoprost (BAK)/ou	3 months	Negative	A, C, E
7	69	M	DED	1st	Type II, os	Latanoprost (BAK)/os	1 year	NA		Negative	B, C
8	78	M	DED, DM	1st	Type I, os	Latanoprost (BAK)/ou, dorzolamide/timolol (BAK)/ou	3.5 years	NA		NA	A,C, E
				2nd	Type III, od	Brimonidine (purite)/ou	8 months	NA			A, C, E
9	74	F	SS	1st	Type II, ou	Carteolol(BAK)/ou	1.5 years	Latanoprost (BAK)/ou	2 months	NA	A, C, F
10	75	F	SS	1st	Type II, ou	Latanoprost (BAK)/ou	5 years	NA		NA	A, C, F
				2nd	Type II, os	Latanoprost (BAK)/ou	1 month	Dorzolamide/Timolol (BAK)/ou	3 days	NA	A, B, D, F
11	70	M	–	1st	Type V, ou	Carteolol (BAK)/ou	6 months	NA		NA	A, B
12	79	M	–	1st	Type II, ou	Timolol (BAK)/ou, Latanoprost (BAK)/ou	5 years	NA		NA	A, C, F
13	79	M	–	1st	Type II, od	Latanoprost (BAK)/od	1 year	Carteolol (BAK)/od	2 months	NA	A, C, E, F
14	96	M	DED	1st	Type II, od Type V, os	Carteolol (BAK)/ou	3 years	NA		NA	A
15	78	M	–	1st	Type II, os	Brimonidine (purite)/os	1 month	Dorzolamide (BAK)/os	8 days	NA	C
16	78	M	DED	1st	Type III, od	Brimonidine (purite)/ou, Dorzolamide/timolol (BAK)/ou	1 month	NA		NA	B
				2nd	Type II, ou	Carteolol(BAK)/ou	1 year	NA		NA	B
17	70	M	DED, DM	1st	Type II, os	Latanoprost (BAK)/ou	2 months	NA		NA	B, C
18	50	M	AD	1st	Type II, os	Brimonidine (purite)/ou dorzolamide/timolol (BAK)/ou	5 years	NA		NA	A, C, F
19	91	M	–	1st	Type II, os	Latanoprost (BAK)/ou	10 years	NA		NA	A

*M* male, *F* female, *DED* non-Sjogren dry eye disease, *BAK* benzalkonium chloride, *NA* not-available, *DM* diabetic mellitus, *SS* Sjogren syndrome, *AD* atopic dermatitis.

^a^Management type: A: preservative-free lubricant, B: TSCL, C: Stopped all glaucoma drugs, D: Stopped last-added glaucoma drugs, E: Shifted to oral anti-glaucoma medications, F: Shifted to preservative-free bimatoprost or carteolol (with BAK).

Exposure durations of long-term eye drops before corneal lesions emerged ranged from one month (case 15, purite-containing brimonidine) to 14 years (case 3, BAK-containing latanoprost). Among the 22 episodes, eight used long-term eye drops for over three months to one year, six used for over 1 year to 5 years, and four used for no more than three months and over 5 years, each. Exposure durations of the last added anti-glaucoma agents before onset of corneal lesions ranged from three days (case 10, second episode, BAK-containing dorzolamide/timolol) to three months (case 6, BAK-containing latanoprost) (Table 2).

### Treatments of anti-glaucoma agents-induced pseudodendritic keratitis

**Figure 3:**
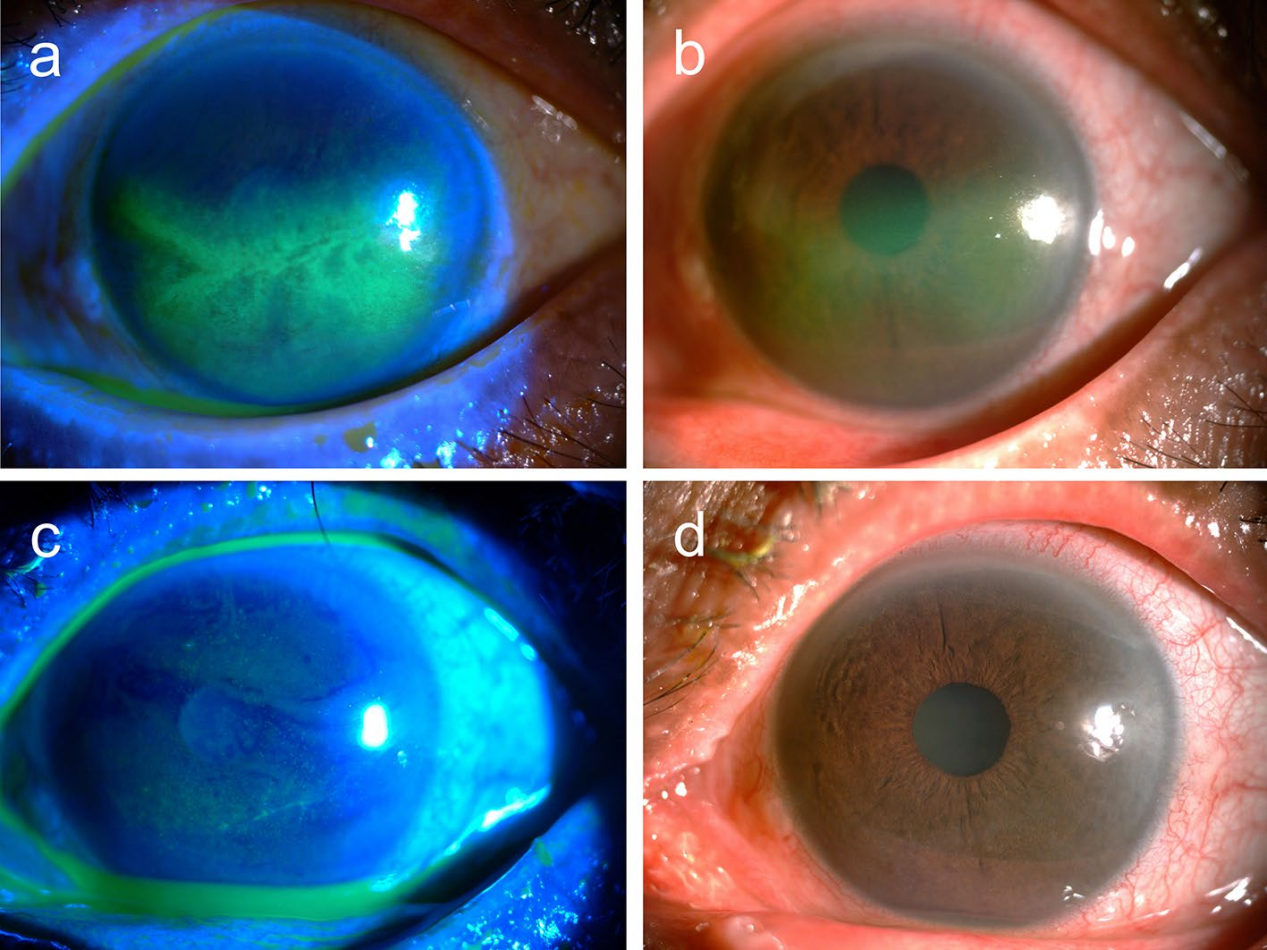
A 75-year-old female (case 10, second episode) with Sjogren syndrome who used latanoprost 0.005% (Xalatan, Pfizer, New York, NY, US, with 0.02% BAK) for one month and dorzolamide 2%/timolol 0.5% combination (Cosopt; Merck & Co., Inc., Whitehouse Station, NJ, US, with 0.0075% BAK) for three days. She presented with type II pseudodendritic lesion from by grouped superficial punctate keratatitis in the left eye with the presumed diagnosis of HSK by the referral ophthalmologist (**a**, **b**). Dorzolamide 2%/timolol 0.5% combination was discontinued, followed by adding preservative free bimatoprost 0.03% (Lumigan, Allergan, Madison, NJ, US) and lubricants. The pseudodendritic lesion resolved within two weeks (**c**, **d**).

**Figure 4:**
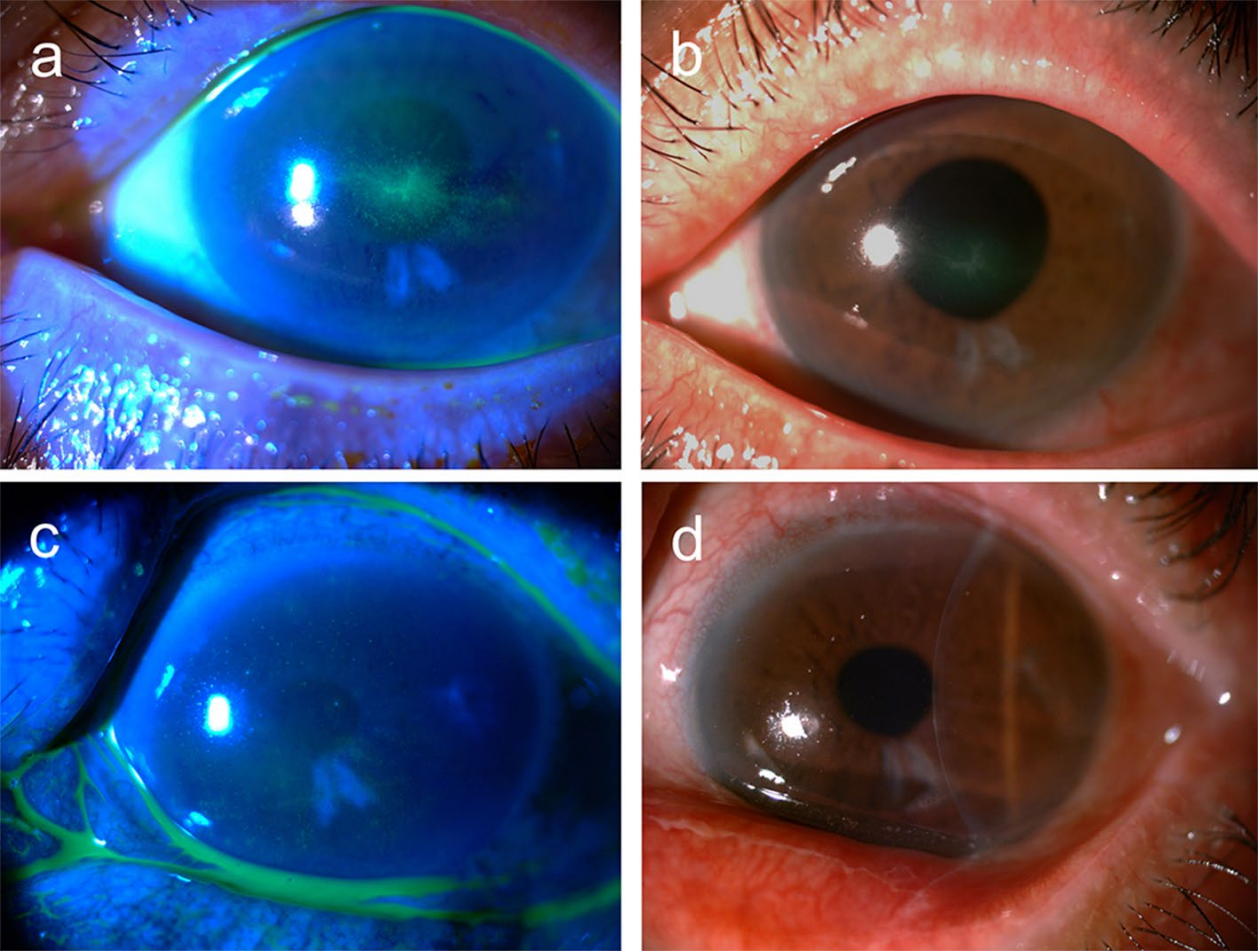
A 69-year-old male (case 7) with dry eye disease used latanoprost 0.005% (Xalatan, Pfizer, New York, NY, US, with 0.02% BAK) for one year. He was referred to our clinic with the presumed diagnosis of HSK, and centrally located type II linear pseudodendritic lesions formed by grouped superficial punctate keratitis was found in the left eye (**a**, **b**). The viral culture was negative. The topical anti-glaucoma agents were discontinued followed by application of therapeutic soft contact lenses. The main lesion resolved within two weeks (**c**, **d**).

Durations of ocular surface symptoms before referral to our hospital ranged from five to 14 days. Of the 19 subjects, five tried acyclovir ointment before referral but showed no amelioration (cases 1, 2, 3, 9 and 13). Following the diagnosis of anti-glaucoma agents-induced pseudodendritic keratitis at our hospital, 15 (68.2%) out of 22 episodes were treated with alternations in anti-glaucoma agents, including change, decrease, or discontinuation of topical medications, while seven (31.8%) reached complete resolution after application of TSCL or preservative-free lubricants without alternating the anti-glaucoma agents (Table 2). Representative external eye photos throughout the clinical course were shown in Figs. 3 and 4.

All pseudodendritic lesions resolved after treatments at our hospital within two months (Table 2). There was no adverse nor unanticipated event. Three patients had recurrent episodes after treatments stopped and the initial anti-glaucoma therapies were resumed. Recurrent episodes relieved after recommence of appropriate treatments.

## Discussion

Topical anti-glaucoma agents, especially prostaglandin analogues, have been proposed to stimulate the recurrence of HSK^18,20,21^. However, corneal toxicity resulted from long-term anti-glaucoma agents usage may also cause pseudodendritic lesions similar to HSK, thus a simple reference guideline to facilitate differentiation is valuable.

Back in 1999, Wand et al. reported three cases of HSK after topical latanoprost therapy, in which one case had viral culture evidence^18^. All cases recovered after discontinuation of latanoprost and one was treated additionally with antiviral agents. Meanwhile, Sudesh et al. reported sterile pseudodendrites caused by latanoprost toxicity^19^. Ekatomatis later reported two cases of HSK diagnosed by immunofluorescence study of corneal epithelium, after using topical latanoprost within three months^20^. Antiviral treatment and discontinuation of latanoprost were applied simultaneously, and elicited lesion recovery. Deai et al. reported positive HSV-1 in tear film through PCR in cases with similar presentation^21^. However, the cases simultaneously applied beta-blocker, a topical agent reported to cause dendritic keratopathy^27^. Notably, a positive PCR result of tear cannot differentiate active HSK and normal viral shedding^30^. Later, several conference reports proposed the existence of non-infectious pseudodendritic keratitis caused by latanoprost, which often manifests as horizontal, opaque, elevated epithelial congregations forming rough branching dendritic figures without terminal bulbs, and broadly surrounded by punctate keratopathy^23,31^. To clarify the association between HSK and prostaglandin analogs, several epidemiological studies were performed, which demonstrated a similar prevalence of HSK between patients treated with anti-glaucoma agents and general public, and a comparable risk of HSK for prostaglandin analogs and other ocular hypotensive agents^22,24^. With the aforementioned findings, the causal relationship between reactivation of HSK and latanoprost should be reconsidered.

In our study, several anti-glaucoma agents, not limited to prostaglandin analogues and mostly BAK-containing, may cause non-infectious pseudodendritic keratitis. Various morphological presentations of the corneal lesions were observed in this study, possibly due to different severities of corneal toxicity, disease stages, and triggering medications with varying active compounds and preservatives. All lesions in our study were located in central to lower corneas. As described above, we classified the lesions into five types, with the majority presenting as linear pseudodendritic lesions formed by grouped superficial punctate keratitis. All linear events (type I and II) were horizontal or curvilinear, which reflected the gravity-dependent accumulation of the toxic topical agents. Notably, different types of lesions were found in some patients with bilateral or recurrent corneal lesions, which supports our presumption that different morphological types of pseudodendritic lesions were all products of similar pathological mechanisms.

The most frequently used medication in our study was latanoprost (0.02% BAK), while other medications included carteolol (0.0075% BAK), brimonidine (0.005% purite), dorzolamide/timolol (0.0075% BAK), dorzolamide, timolol (0.01% BAK), pilocarpine (0.01% BAK). Although it is challenging to conclude whether the corneal toxicity was caused by the active compounds or the preservatives in these anti-glaucoma agents, BAK was found in most of the candidate triggering medications. The exposure durations of long-term and last-added anti-glaucoma agents before the onset of pseudodendritic lesions were one month to 14 years, and three days to three months, respectively. Contrary to the wide range of long-term drug application durations, the patients seemed to notice the symptoms more rapidly when new topical agents were added. Hence, it is crucial for clinicians to be attentive to those receiving long-term therapy, since the prolonged medication use and lack of significant pharmacological changes may easily lead to negligence of the drug toxicity. For patients receiving long-term medications and prescribed with new anti-glaucoma agents, frequent follow-ups should be arranged initially. While it is known that the ocular toxicity of BAK is dose-dependent^13,32^, the exact amount of BAK exposure needed for pseudodendritic keratitis development is yet to be determined. In addition, since most of our enrolled patients were older than 65 years old and had underlying conditions prone to ocular surface diseases, including DED, DM, SS, selection of anti-glaucoma agents and application duration should be carefully determined in patients with these characteristics.

The main challenge of our study is the confirmation to exclude HSK. No patient in our study had known history of HSK or herpes zoster infection. Cases 1–7 received viral culture and demonstrated negative results, thus laboratory viral tests were not performed on the others. About half of the patients suffered from bilateral corneal lesions, which may indirectly rule out the possibility of HSK, since HSK rarely cause bilateral involvement in immunocompetent individuals. Interestingly, in the other patients with unilateral lesions, most received bilateral anti-glaucoma treatment with same medications. The reason for the observed unilateral involvement in these cases may be related to the asymmetrical dosage application by patients or the different baseline characteristics of each eye, for example, various degrees of dry eye disease severity. This observation requires further investigations.

The orientation and location of the pseudodendritic lesions implied the underlying gravity-dependent toxin accumulation. This is different from HSK, in which no preference on the location and orientation of the dendrites were documented. Disregarding the types of morphology, all lesions in this study demonstrated lack of the characteristic terminal bulbs and central fluorescein stain usually found in HSK. In addition, most lesions were surrounded by grouped SPK. The unique presentations can be considered a clue for differential diagnosis. No antiviral medication was prescribed to any patient after referral, and the corneal lesions mitigated under decreased anti-glaucoma drug burden or ocular surface protection with TSCL or lubricants. Satisfactory therapeutic responses after these measures further indicated the non-infectious entity of these lesions. Although other causes including healing epithelial defect, recurrent erosion syndrome^33,34^, soft contact lens wear^35^, neurotrophic ulcer^36^, acanthamoeba keratitis^37^, systemic tyrosinemia^38,39^, and Thygesons’s superficial punctate keratitis^40^ may also cause pseudodendritic lesions, clinical presentations and past history of our patients suggested these etiologies to be unlikely.

Treatments for anti-glaucoma agents-induced pseudodendritic keratitis followed previous guidelines^41^. Discontinuation or alternation of anti-glaucoma agents, application of preservative-free lubricant or TSCL were the main approaches. Time to recovery varied, but complete resolution was reached within two months in all patients. Ultimately, a stepwise approach is needed to determine the most suitable substitute anti-glaucoma agents that balances between the adverse effects and the therapeutic effects, and oral anti-glaucoma agents may be considered in the meantime. Since most cases in our study applied BAK-containing anti-glaucoma agents, changing to non-BAK-containing or preservative-free anti-glaucoma agents may be eligible. Fixed-combined medication, which can decrease the overall drug burdens, can also be beneficial for reducing drug toxicity. However, some patients in our study were already using fixed-combined medication, either as monotherapy or in combination with other anti-glaucoma agents, before the occurrence of the pseudodendritic lesions. After treatments, there were three patients with recurrence. Patients 8 and 16 resolved completely after removing all presumed triggering anti-glaucoma medications, but recurred when applying new medications. Patient 10 also achieved complete resolution in the first episode, but recurred when resuming latanoprost. Hence the second episodes of these patients should be viewed as separate incidents and required independent evaluation. All three patients had past ocular history of DED or SS and two of them used anti-glaucoma agents for over three years before the first episodes, which might have compromised the ocular surface integrity and led to greater susceptibility to the second episodes.

There are some limitations in this study. First, the retrospective nature and limited case number are the main drawbacks. Second, despite that we provided a comprehensive diagnosis guideline, lack of laboratory test confirmation in the enrolled cases is still a concern. Third, corneal sensitivity test was not performed to rule out the possibility of neurotrophic keratitis. Both HSK and chronic use of topical medications containing BAK can cause impaired corneal sensation^14^. Since most patients have applied anti-glaucoma agents for a while and decreased corneal sensitivity might have already occurred, this test may not be optimal to differentiate HSK and anti-glucoma agents-induced lesions, as both conditions are characterized by abnormal corneal sensitivity. Hence, corneal sensation was not tested as it would be difficult to differentiate the exact cause if decreased result was observed.

In conclusion, our study described a non-infectious pseudodendritic keratitis caused by anti-glaucoma agents, which can be easily misdiagnosed as HSK. However, the patient’s medical history and the unique morphological characteristics of the corneal lesions can help distinguish them, even without microbial confirmation. These features include horizontal-linear or curvilinear, instead of vertical-linear, orientations of the lesions with predominant locations at the lower to middle parts of corneas, and the absence of terminal bulbs. Physicians should also keep in mind that, in addition to linear lesions, anti-glaucoma agents-induced pseudodendritic keratitis can occasionally present as satellite lesions and geographic ulcers. Discontinuation of the triggering medication, decreasing drug burden, and providing ocular surface protection are sufficient for complete resolution of these lesions, while antiviral therapy is not recommended. Most importantly, clarification of medication history and disease course and observation of the corneal lesions are keys to identify this disease, which were included in the reference guideline provided in our study.

## Data Availability

All data relevant to the study are included in the article.
